# Acknowledgement and the paradox of tragedy

**DOI:** 10.1007/s11098-015-0495-0

**Published:** 2015-05-08

**Authors:** Daan Evers, Natalja Deng

**Affiliations:** Faculty of Philosophy, University of Groningen, Oude Boteringestraat 52, 9712 GL Groningen, The Netherlands; Faculty of Philosophy and Faculty of Divinity, University of Cambridge, Sigdwick Avenue, Cambridge, CB3 9DA UK

**Keywords:** Paradox of tragedy, Acknowledgement theory, Susan Feagin, Jonathan Lear

## Abstract

We offer a new answer to the paradox of tragedy. We explain part of the appeal of tragic art in terms of its acknowledgement of sad aspects of life and offer a tentative explanation of why acknowledgement is a source of pleasure.

## Introduction

In this paper, we offer a new answer to the paradox of tragedy. We propose to explain part of the appeal of tragic art in terms of its acknowledgement of sad aspects of life. It is organized as follows: we define the problem in Sect. 1 and develop our solution in Sect. 2. Section 3 discusses objections to the idea that acknowledgement is a source of pleasure. Section 4 answers objections to the idea that pleasure explains the appeal of tragic art and Sect. 5 offers a tentative explanation of the fact that we derive pleasure from acknowledgement at all. Section 6 compares our view to Jonathan Lear’s work on katharsis, and Sect. 7 answers some further objections.

## The paradox of tragedy

The problem we will address can be characterized in either one of two ways. The first (after Smuts 2007, 2009) is this: why do people pursue art that evokes negative emotions, when they tend to avoid things that evoke such emotions?^1^ The emphasis here is on the disagreeable nature of certain mental states. The second characterization emphasises the disagreeable nature of their causes (which are also, typically, their objects): why do we appreciate tragic events in art when we don’t appreciate tragic events in life?

We will start with the first question: why do people pursue art that evokes negative emotions, when in general they avoid things that evoke such emotions? The emotions in question include sadness, pity, heartache, feelings of loneliness, disappointment, guilt, shame and regret. Even certain kinds of fear, disgust, shock, distress, anger and indignation are relevant. What is negative about these emotions is their tone or phenomenal quality: it *feels* bad to have them.

In this form, the paradox applies not only to tragedy in a broad sense, including tragedies (in the narrow sense), melodramas, sad songs and so on; but also to horror. Smuts speaks broadly of ‘painful art’, a term intended to cover all these kinds of art.

However, we will restrict our attention to tragedy in the broad sense, which we will simply call ‘sad art’. The classification is only rough and ready, and turning it into a definition may be problematic. But we hope that the above open-ended list is a clear enough guide. Note that the class of relevant works is not restricted to works with ‘unhappy endings’, as Feagin says in (1983). Many works with happy endings evoke negative emotions in certain scenes, or even on the whole. Certain kinds of comedies, or parts thereof, can be relevant too. The BBC series *The Office* is a good example.

Although horror typically evokes negative emotions which are also relevant to sad art (such as fear and disgust), we think its appeal is likely to differ from that of tragedy: what we like about scenes with supernatural monsters is probably different from what we like about scenes in concentration camps. But to the extent to which horror involves what we intuitively think of as sad or tragic elements, our theory applies to our appreciation of these too.

To be clear, in calling the relevant kind of art ‘sad’, we don’t mean to imply that sadness is the only kind of negative emotion under discussion. We are merely focusing on (mostly) non-horrific art that arouses any of the above-mentioned negative emotions. For brevity, we will sometimes refer to these emotions simply as ‘sadness’ (thereby stretching the ordinary meaning of the term).

You may wonder whether the paradox is genuine. It might be objected that the first characterization rests on a false assumption: people may avoid the *causes* of negative emotions in real life, such as actual loss, but at times seek out and even enjoy the emotions themselves, such as sorrow or sadness.^2^ One might even hold (with Dubos as presented by Hume in *Of Tragedy*) that feeling any strong emotion, whether negative or not, is desirable; feeling strongly is simply its own reward. This is also Smuts’s view.

But how plausible is it that we tend to pursue or enjoy being sad? It seems at least as plausible that we often prefer feeling better over prolonging or deepening negative emotions. That we *sometimes* pursue such emotions for their own sake is compatible with the first formulation of the paradox. All one needs for that problem to arise is that we *tend* to avoid things that make us sad, where this is due not merely to their causes. This seems to us a reasonable assumption. Further support comes from the fact that some people *do* avoid sad art. But imagine they experienced no negative emotions in response to it. Would they have the same reasons to avoid it? This seems implausible. We tend to avoid potential causes of grief at least in part because they cause us *grief*.

This brings us to the second characterization of the paradox of tragedy: why do we appreciate sad events in art in a way in which we don’t appreciate sad events in life?^3^ Note that the question is not why we generally don’t appreciate sad events in life; that much is obvious, we don’t appreciate actual loss or injury. The question is rather why we do (positively) appreciate sad events in art. That question is not answered by the fact that art involves only fictional losses.

To this it might be objected that we sometimes do appreciate negative events in life. Some of us are prone to *Schadenfreude* and most of us to *Schaulust*: people enjoy looking at car wrecks, bullfights, and crucifixions.^4^ However, we think there is a kind of appreciation of sad art that does not consist in feelings of this kind. First, it seems phenomenologically false that our appreciation of tragedy is mostly due to *Schadenfreude* or *Schaulust*. Second, if it was, it would be hard to see why anyone would think of such art as morally edifying.

Another objection to the second formulation of the paradox is that in art too, we often deplore sad turns of events: for example, we don’t want Desdemona to die. So there is no discrepancy between life and art in this respect.^5^ This objection highlights the fact that our appreciation of sad art need not be an appreciation of the suffering of fictional characters (indeed, our own solution suggests a different cause). But there seems to be a discrepancy between sad art and life at least in the sense that we seem to appreciate tragic art partly for its depiction of sad events. There seems to be no analogue of this kind of appreciation in life.

## The acknowledgement theory

We think both questions involved in the paradox can be answered with reference to the fact that sad art *acknowledges* sad aspects of life. Take Robert Herrick’s *Gather Ye Rosebuds*, for instance. This familiar poem starts as follows:

‘Gather ye rosebuds while ye may,

Old time is still a-flying:

And this same flower that smiles to-day

To-morrow may be dying’

This verse draws attention to the passage of time, the loss of youth and the inevitability of death. Why should we enjoy it? Several reasons spring to mind. For instance, we may like its language or the *way* it talks about its subject matter. However, it seems wrong that our appreciation is only due to formal features of the work. We also appreciate it because of *what* it talks about, even if that’s sad. We would like to suggest that our appreciation of its sad content is due to the fact that the poem *acknowledges* transience and mortality.

Acknowledging something ordinarily involves recognizing it, giving it its due, giving it credit, honouring it, or doing it justice. A work does this by the very selection of its subject matter and by the stance it takes towards it: it provides a standing occasion to respond with negative emotions to certain events, signalling that they are significant.

When we speak of the ‘significance’ of sad aspects of life, this obviously cannot be understood in terms of positive value. The loss of a child is a terrible thing. But *that* life contains such loss matters or is significant to us in the sense that we find it an aspect of life worth mentioning, remembering and exploring in art. ‘Acknowledgement’ seems to us to capture this phenomenon well. As we said, acknowledging involves recognizing, giving credit, honouring, or doing justice. We think that sad art does just this for its subject matter. In this respect, works of sad art have much in common with monuments to real life tragedies. The difference is that since sad art typically touches on universal themes, it ‘commemorates’ not only specific events, but general aspects of life.^6^

We think this view accurately captures a source of appeal in many works of sad art. Consider *A Serious Man* by the Coen brothers. The film tells the story of a Jewish professor who suffers a series of professional and personal misfortunes. Hoping to find relief of feelings of absurdity and meaninglessness, he consults a number of rabbis who don’t offer any answers. Part of the appeal of these sequences lies in their acknowledgement of feelings of meaninglessness and religious doubt as significant events in people’s lives.^7^

Another example is a photograph by Kevin Carter that shows a starving child and vulture, apparently waiting for the child to die. The subject matter here is grim, heart-wrenching, shocking. Few would enjoy a scene like this in life. But we think there is a sense in which one enjoys looking at the photograph. The reason lies in what it represents. It represents a point of view from which the plight of this child, and more general facts about the depths of human suffering, are acknowledged.

In Sect. 1, we distinguished two characterizations of the paradox of tragedy. The first was this: why do people pursue art that evokes negative emotions, when in general they avoid things that do this? The second was different: why do we appreciate sad events in art in a way in which we don’t appreciate sad events in life? Our answer to the second question suggests our answer to the first: we appreciate sad events in art because we take pleasure in the work’s acknowledgement of sad aspects of life. We don’t appreciate such events in life because they don’t involve acknowledgement at all. They are simply bad events. The first question is then answered as follows: we don’t avoid negative emotions in art because we derive pleasure from acknowledgement.

## Do we derive pleasure from acknowledgement?

Our theory is in trouble if acknowledgement is not a credible source of pleasure to begin with. Some people object to our comparison of sad art with monuments or ritual commemorations of real-life tragedies. Most people do not experience funerals as pleasurable, even though they involve acknowledgement. This threatens our thesis that acknowledgement accounts for the fact that we appreciate sad content in art.

It is true that attending funerals or commemorations is in many ways unlike consuming sad art. However, we think that there is one respect in which the two experiences are alike, and that this does important work in explaining our appreciation of sad art.

Some real-world commemorations and most funerals we attend involve sad events that we are personally involved in. For this reason, they can occasion acute grief and anguish, which can make it impossible for the acknowledgement involved to give rise to any pleasure. However, that does not mean that acknowledgement cannot be a source of pleasure on occasions when one is not involved in this highly personal way.

It is not implausible that being moved by something is a form of pleasure. This often occurs both in art and funerals. Furthermore, we do at least *value* the acknowledgement of events we think are sad and significant. Witnessing the occurrence of something one values is typically a positive experience.

Some people object that news reports acknowledge sad events without eliciting pleasure. However, news reports typically don’t *acknowledge* sad events in our sense of the term. Art acknowledges sad events by representing them *as* sad and as significant. It does not merely *report their occurrence*, which is the primary aim of the news. Furthermore, news items typically don’t involve the sort of detail normally present in narrative artworks. Art rouses our emotions by getting us invested in the fate of particular protagonists. So the level of emotional involvement is usually much greater. This emotional involvement explains why we typically find the acknowledgement of sad events in art satisfying: we (are made to) care more about the subject matter.

Of course, news reports (or documentaries) can be very detailed too, and they can also involve acknowledgement in our sense. But when they do, it is no longer implausible that they give rise to the pleasure from acknowledgement.

## The pleasures of tragedy

The acknowledgement theory says that people derive pleasure from the fact that certain aspects of life are acknowledged in works of art, and answers the question why we pursue tragic art with reference to this pleasure. This means that Smuts (2009) would classify the view as a hedonic compensation theory. According to such theories, the pain experienced in response to sad art is compensated for by pleasure. Smuts raises an objection to these views. According to him, compensation theories wrongly portray art’s power to evoke negative emotions as a *problem*, something that requires compensation and is not itself valuable.

We think this criticism is just only if taken in a certain way. Since we don’t generally appreciate events that induce negative emotions in life (Sect. 1), it is not wrong to look for some feature of art *distinct* from its power to evoke negative emotions which explains our appreciation. What would be wrong is to look for a feature *notessentially tied* or related to the sadness of depicted events.^8^ For example, it would be wrong to suggest that we appreciate *Othello* because its comedic moments or set design make up for the sad story. But it needn’t be wrong to identify a pleasurable feature essentially connected to the sad nature of depicted events. Such a feature can explain why we appreciate such events in art but not in life.

The acknowledgement theory does forge an essential link between our appreciation of sad art and the sad nature of events depicted in it. The theory claims that we appreciate the acknowledgement of *sad* events.

The claim that we derive pleasure from the acknowledgement of sad events does not require that pleasure occurs at every point during the experience. This is particularly relevant in the case of narrative works of fiction. In the final scene of *The Great Silence*, the hero is shot by immoral bounty hunters, along with everyone he was trying to save. When the shooting occurs, negative emotions dominate, so it would be wrong to say that we immediately enjoy the scene. But we can nonetheless derive pleasure from it, due to the fact that it acknowledges the injustice of the act. (The scene is actually followed by a statement commemorating a fictional massacre in the USA.) This pleasure may only occur at a point where the viewer is not completely overwhelmed by negative emotions.

It is also not required that the pleasure from acknowledgement has greater felt intensity than the negative emotions which are triggered by the work. This kind of pleasure is almost certainly not as ‘violent’ as the shock of seeing people slaughtered. We don’t think this entails that the depiction of sad events is a stronger deterrent than it is an attraction. The weight of pleasures and pains is not always a function of their felt intensity. For example, mountain climbing can be a pleasurable experience, even though it involves phenomenally strong pains and exhaustion. Conversely (though perhaps more controversially), phenomenally strong pleasures needn’t make an experience pleasant on the whole. Examples may be guilty pleasures (such as eating something that interferes with one’s diet), or strong drug-induced pleasures while wishing one hadn’t taken the drug.

It is important to remember that our theory only pertains to our enjoyment of a work insofar as it involves sad content. The question why we enjoy this aspect of a work is distinct from the question why we enjoy the work on the whole. The latter may be answered by reference to many different features: visual beauty, poignant dialogue, surprising construction, as well as the acknowledgement of certain tragic things. But our appreciation of its sad content is specifically explained by the pleasure from acknowledgement.

## A tentative explanation of the pleasure from acknowledgement

One may wonder *why* the acknowledgement of sad events would be a source of pleasure. We only offer a tentative explanation.

Susan Feagin is famous for a theory according to which we appreciate the depiction of sad events in art because it would give rise to a meta-response (1984). Although we experience negative emotions in response to art (the direct or first-order response), we take pleasure in the fact that we *have* such emotions. For instance, we take pleasure in the fact that we are the kind of beings who feel pity for Oedipus. Feagin often writes as if the reason for this is that we evaluate our reactions as morally appropriate or laudable. For example, she writes that

‘We find ourselves to be the kind of people who respond negatively to villainy, treachery, and injustice. This discovery, or reminder, is something which, quite justly, yields satisfaction.’ (Feagin 1983, p. 98)

This idea invites the objection that our appreciation of sad art does not seem akin to moral satisfaction with one’s own first-order emotions. But some claims that Feagin makes point to a different source of pleasure. Take the following passage:

‘In a way it [the fact that we have certain emotional responses] shows what we care for, and in showing us we care for the welfare of human beings and that we deplore the immoral forces that defeat us, it reminds us of our common humanity. It reduces one’s sense of aloneness in the world, and soothes, psychologically, the pain of solipsism.’ (Feagin 1983, p. 98)

Here Feagin suggests that the reason why we enjoy sad art is that it reminds us that we are not alone in the way we feel about the world, that we share certain responses. We would recognize ‘that there can be a unity of feeling among members of humanity’ (Feagin 1983, p. 103).

Suppose Feagin is right that experiencing sad art brings with it an awareness not only of the sad (terrible, unjust etc.) events portrayed, but also of the fact that other people, including the makers and other potential viewers, are sympathetically aware of these events. Might this not in itself be comforting, and ‘remind us of our common humanity’—not because it shows that we are moral creatures, but simply because it shows that others, like us, are aware of certain events and share our responses to them?

It’s often comforting to know that other people too are aware of certain sad aspects of life that one encounters or can imagine encountering. And it’s comforting to know that they feel the same way about them. In this way, our appreciation of sadness in art might derive from an awareness of shared sympathetic responses, even if what we appreciate has nothing to do with their moral quality.

The result would be a modified version of the meta-response theory: the pleasure we derive from tragedy is a response to the fact that others respond in similar ways. But there are problems with this theory too.^9^

When consuming and enjoying a work of sad art we do not seem to reflect on our responses to its content. Similarly, we do not seem to reflect on the fact that others share our responses to the work. Indeed, being reminded that there are other actual viewers (say, next to us in the cinema or gallery), or even that there are other potential viewers, may detract from our enjoyment. Nor is it plausible that our enjoyment is due to reflection on the *maker* of the work. We can enjoy a work of art without thinking about the artist.

However, we may be able to derive pleasure from the fact that *the work* shares certain responses with us. By presenting events *as* sad and as significant (i.e. by acknowledging their sadness and significance), the work constitutes a perspective on its content distinct form the viewer. Perhaps the pleasure from acknowledgement is due to the fact that we share this perspective *with the work*. In order for this to happen, all you have to do is focus on the work, not on other people.

One may still feel that this explanation of the pleasure from acknowledgement is implausible. It seems to entail that appreciation of a work of sad art involves (a) consciousness of the fact that the work has a certain view about certain aspects of life, (b) consciousness of the fact that we ourselves have this view and (c) consciousness of the fact that we share this view with the work of art. This seems as cognitively demanding and phenomenologically inaccurate as Feagin’s theory.^10^

We are not convinced of this, however. In consuming works of art our attention is primarily focused on the work itself. But since part of understanding a work of art involves understanding its stance towards its subject matter, (a) is unproblematic. Moreover, (b) and (c) are not required for what we say. Your views may be causally responsible for emotional effects even if you don’t consciously represent them to yourself. If you hear someone make a racist statement, you may feel irritation. You would not feel this unless you were against racism yourself. But in order for your irritation to arise, you needn’t have been thinking consciously about your own commitments. In the same way, you can enjoy the expression of a sentiment because you share it with that person, even if you don’t consciously reflect on the fact that you have this sentiment, or that you share it.

But even if this were false, and it wasn’t possible to enjoy the fact that a work shares your perspective without being conscious of the fact that you share it with the work, this need not be as implausible as might appear at first. One’s absorption in a work of art is seldom so great as to allow no reflection on the work and one’s own responses. This is supported by the fact that most people who read a book or watch a film are on some level aware *that* they are reading a book or watching a film. So our enjoyment of a work may in part depend on the interplay between our focus on the work and reflection on it as well as on ourselves.

There are of course other objections. For example, sad art often deals with topics one has no direct experience with (such as the loss of a child). In what sense is there a ‘pain of solipsism’ here?^11^

Here one might note that sad art often deals with universal themes of life. But even when it doesn’t, it may help us understand what it is like to be involved in unfamiliar situations. This enables us to relate to suffering remote from our own lives. So although we didn’t feel alone with respect to these particular events prior to our engagement with the work, we can still appreciate the fact that they have been acknowledged by engaging with the work.

So it is not obvious that the explanation of the pleasure from acknowledgement in terms of sharing a perspective is mistaken. However, we do not insist on it. We are primarily committed to the idea that we appreciate the depiction of sad events in art because we derive pleasure from the acknowledgement of sad aspects of life. We are committed to this even if our explanation of that pleasure is wrong, or if no informative explanation is to be had (i.e. if it is a brute psychological fact that we experience pleasure from acknowledgement).

## Jonathan Lear on katharsis

The idea that pleasure is derived from the acknowledgement of sad aspects of life bears similarities to work by Jonathan Lear on Aristotle’s notion of katharsis (1988).^12^ Lear shares our view that sad art affords a kind of pleasure (p. 302), and offers several distinct ideas about the reasons why.^13^ All of these relate in one way or another to the fact that sad events are significant to us, although the details differ. The main idea is that there is *relief* involved in experiencing fear and pity in response to fictional tragedy (pp. 323, 325). This relief would be due to the fact that fiction affords a context in which one can appropriately experience emotions for which there is little place in everyday contexts. The emotions in question concern remote tragic possibilities in our own lives. The fact that such possibilities are unlikely explains why it is inappropriate to indulge in these emotions in everyday contexts. However, the fact that human life is nevertheless vulnerable to tragic events is ‘a possibility we must live with’ (p. 324), and our standing belief that they might befall us ‘does exert some pressure on our souls’ (p. 323). The latter seems to explain why it is a relief to be able to experience them in response to art.

One natural reading of this view is in terms of repression or ‘pent-up emotions’ (p. 325).^14^ Since at least relatively virtuous people respond appropriately to what the situation demands, such people will not indulge in fear with respect to remote possibilities. Nevertheless, even they feel some “pressure” arising from the standing belief that tragic events might happen. This pressure can be thought of as a tendency to experience fear and related emotions anyway, and art as an outlet for the tendency that is otherwise silenced or overruled by other dispositions. (The background assumption is of course that it is not inappropriate to feel pity for protagonists and fear for one’s own life in the theatre, say, where certain possibilities are explicitly addressed.)

If this is indeed part of Lear’s view, ours is distinct in certain respects. Whereas Lear would say that sad art gives rise to pleasure because it is an outlet for repressed emotions, this is no part of our view. We think it gives rise to pleasure because we enjoy the acknowledgement of sad aspects of life (and our tentative explanation of the latter does not involve repressed emotions either).^15^

There is some reason to doubt that relief is the right way to describe the pleasure taken in sad art. First, insofar as relief is the result of not being able to experience certain emotions in other contexts, we should note that some art is about sadness one may have encountered in one’s own life (such as a failed relationship). There is little reason to think one’s enjoyment of a work would be diminished if one had recently experienced similar emotions in a real-life situation (in fact, we think it might be heightened). Second, it is not clear that the idea of pent-up emotions with respect to tragic possibilities is psychologically plausible.

One may also question the idea that the reason why we enjoy sad events in art relates to a tendency to experience a kind of fear with respect to sad events *that might happen to ourselves*. Lear defends Aristotle’s view that we experience pleasure from tragedy only insofar as we take ourselves to be sufficiently like the protagonists to believe that what happens to them might happen to us too. Although this point is plausible, it needs to be interpreted with care. One reason the belief might be necessary is that we take pleasure in confronting or releasing a fear whose object is oneself. This appears to be Lear’s suggestion. It is not obvious that one’s enjoyment of King Lear, say, would (in part) consist in releasing a fear about losing one’s own sanity. But another reason the belief might be necessary is that it enables us to engage emotionally with the protagonists and story to a greater degree. The latter is compatible with the idea that we don’t ordinarily feel a kind of pressure from the possibility of sad events in our own lives, and leaves the question why we enjoy work with tragic subject matters hanging.

Our view is that sad art gives rise to pleasure because we enjoy the acknowledgement of sad aspects of life. Sometimes this enjoyment may be due to the fact that we are familiar with certain kinds of suffering from our own lives, or fear that suffering ourselves. But this is not essential to our view. What is essential is that we *care* about the depiction of sad events, and we do this because we find them significant. One can find sad events significant and worthy of discussion without fearing that they might happen to oneself.

However, Lear also offers two reasons why sad art would be a source of consolation, and he describes these as explanations of ‘the content of our relief’ or ‘what our relief is about’ (p. 325). The first reason why tragic art would be consoling relates to Aristotle’s requirement that the events in tragic theatre must be plausible or necessary: they ‘must occur on account of one another’ (p. 325). This offers the consolation that even though bad events happen, at least they are intelligible: they do not ‘occur in a world which is in itself ultimately chaotic and meaningless’ (p. 325). Lear thinks it is part of the intelligibility requirement that if bad things happen to a good person, they are the result of a mistake which ‘rationalizes his fall’ (p. 325), rather than some accident or natural disaster.

The second reason why tragic art would be consoling consists in the fact that it shows that ‘humans remain capable of conducting themselves with dignity and nobility’ (p. 326), even when they are responsible for their own misfortune.

Although interesting, we doubt that these explanations of the nature or object of the pleasure experienced in response to tragic art are generally valid. With respect to the first: it seems possible to experience the kind of pleasure typical of sad art in response to works that portray the world as meaningless, or in which bad events do occur as a result of natural disaster. With respect to the second, it seems possible to experience the relevant kind of pleasure in response to works that portray the onset of dementia or madness, in which a loss of dignity may be involved. For these reasons, we prefer to say that we take pleasure in sad art because it acknowledges sad aspects of life.

## Further objections

In this final section, we discuss some further objections to the acknowledgement theory.

1. Acknowledging aspects of life is not unique to works of *sad* art. But if so, how can this be an answer to the paradox of tragedy?

It may well be that acknowledgement is not confined to sad art. But if both positive and negative aspects of life can be acknowledged, this does not undermine the claim that our enjoyment of sad art is partly due to the acknowledgement of *sad* features. In fact, our theory gains further credibility if acknowledgement plays a larger role. And it does seem plausible that the appeal of e.g. (non-tragic) love poems is partly due to their acknowledgement of the importance of love in our lives.^16^This point is compatible with our explanation of why a work’s sad content is essential to the compensatory pleasure (Sect. 3). It is essential to being pleased by the acknowledgement of *sad* aspects of life that they are sad and presented as such.

2. Doesn’t the acknowledgement theory presuppose an unrealistic degree of interest in moral issues on the part of consumers? Most people are not particularly concerned about sad aspects of life, at least not in a way which spurs them on to action. So why should the acknowledgement of such aspects be a source of pleasure for most people?

First, a lot of sad art deals with universal aspects of life that are not candidate targets for activism (jealousy, love, failed aspirations). Second, since such themes are familiar to everyone, it does not take an altruistic nature to care about them. Third, engaging with a work of art makes certain things emotionally salient which one needn’t be concerned with beforehand or afterwards. But while you care, you can care for their acknowledgement.

3. Some art has disturbing or shocking subjects, such as Ronald Ophuis’s paintings of physical, sexual and psychological violence. Do we really take pleasure in such works?

It is important to keep in mind that we don’t appreciate the acknowledgement of sad events because we value them; violence is not valuable. But the fact *that such violence occurs* is worth acknowledging in art. This is the source of pleasure. However, there probably are cases where strong negative emotions leave no room for any kind of pleasure (this parallels our earlier remarks about funerals in which one is personally involved). In such cases, acknowledgement may still explain why we value art with these subject matters (if not why we enjoy it). But we think that most sad art does involve some pleasure.

Also, some works of art with disturbing subject matter may not take a negative attitude towards it, and thereby fail to acknowledge its sadness (novels by Marquis de Sade, for instance). Our theory identifies one reason why we don’t enjoy such works.

4. Bad art can acknowledge sad aspects of life as well. But it is not plausible that we derive much pleasure from it.

We don’t think that objective quality is relevant (it can hardly be claimed that people don’t derive pleasure from melodramas). But it is plausible that we don’t derive much pleasure from art which we strongly dislike, even if it acknowledges sad events. This, however, does not clearly undermine our theory. First, one’s dislike of a work may overshadow positive emotions. Second, in order for the pleasure from acknowledgement to occur, one has to be engaged with the work in such a way as to trigger the relevant negative emotions. This is less likely if you strongly dislike the work. But if these emotions are absent, your level of (occurrent) concern for the subject matter will be low, which means you are less likely to derive much pleasure from acknowledgement.

## Conclusion

We have formulated a new answer to the paradox of tragedy. We proposed that our appreciation of sad art is due to the fact that it acknowledges the sadness and significance of certain aspects of life. We think this is a source of pleasure. It is not entirely clear *why* it is a source of pleasure, and we offered a tentative answer: the pleasure from acknowledgement may be due to a sense of shared awareness of the sadness and significance of events depicted in a work. This sense would be due to the fact that the work itself constitutes a perspective on its content. If this explanation is correct, then the pleasure from acknowledgement is similar to the pleasure derived from an awareness of not being alone in one’s feelings and emotions.

## Footnotes

1 Like Smuts, we will sidestep the issue of whether we have genuine emotions in response to art. All that is needed for the paradox is that we have things very much like emotions, some of which are unpleasant, in response to art.

2 Thanks to Rob van Gerwen for raising this issue. See also Kendall Walton (1990), pp. 257–258.

3 Putting the question in terms of ‘appreciation’ allows us not to prejudge whether pleasure is involved. As we explain in Sect. 3, our own solution does posit pleasure as part of our response to tragedy.

4 Christopher Williams (1998) even thinks this dissolves the paradox of tragedy.

5 This arguably raises another problem about tragedy (which relates to more general problems about our imaginative engagement with fiction): on the one hand we want tragic fictions to include various sad events; on the other hand, we react in ways which seem to show that we do not want those events to occur. See e.g. Gregory Currie (2010) and Amy Kind (2011).

6 Which is not to say that monuments to real life tragedies cannot serve more general commemorative purposes.

7 People inclined to object that *A Serious Man* is not a good example because it is a comedy should ask themselves whether they really believe that the appeal of the film is wholly due to its comedic features and not at all to its tragic content. That seems clearly wrong to us.

8 Alex Neill makes a somewhat similar point, though as part of a critique of compensation theories. See Neill (1992a), p. 58 and Neill (1992b), p. 153. For the opposite viewpoint see Packer (1989).

9 In considering this objection we benefited from discussions with Tom Stern.

10 Thanks to Matthew Kieran for pressing this objection.

11 Something like this objection was raised by Tom Stern.

12 This was pointed out to us by an anonymous referee for this journal. We are grateful for the reference.

13 In what follows we will often write as if the view Lear attributes to Aristotle is also his own. This is not certain, but Lear is clearly sympathethic to the view.

14 Although this does raise the question whether it is compatible with being a virtuous person that one has to repress emotions. It is important to Lear to preserve the idea that katharsis can also be experienced by the virtuous, as Aristotle seems to claim as much himself (1988, pp. 302, 303).

15 An anonymous referee for this journal has suggested that the pleasure experienced as a result of tragic fiction might, for Lear, be (partly) due to the fact that fiction allows us to confront and contemplate general (and in this case sad or disturbing) truths about life. Contemplating such truths would be pleasant because all people would delight in knowing or learning. However, Lear explicitly rejects the idea that the peculiar pleasure of tragedy is cognitive (1988, p. 307). Like him, we find it hard to believe that this pleasure is (mostly) due to the fact that contemplating truths in general is pleasant. It does not seem true to the phenomenology of art experience and (appears to) ignore the peculiarity of the enjoyment of sad art. (The latter is of course debatable.)

16 Putting it like this may make the appeal of love poems sound too intellectual, as if it merely involved the recognition of a certain abstract truth about human life. This is not what we intend. We take pleasure in the acknowledgement of specific emotions which the poem evokes and which we may recognize from personal experience.
